# HMGB1 promotes HCC progression partly by downregulating p21 via ERK/c-Myc pathway and upregulating MMP-2

**DOI:** 10.1007/s13277-015-4049-z

**Published:** 2015-10-24

**Authors:** Yanmei Chen, Chengzhao Lin, Yang Liu, Yan Jiang

**Affiliations:** 1 Institutes of Biomedical Sciences, Shanghai Medical College, Fudan University, Yixueyuan Rd 138, Shanghai, 200032 China; 2 Department of Chemistry, Fudan University, Shanghai, China

**Keywords:** HMGB1, HCC, MAPKs, c-Myc, p21, MMP-2

## Abstract

High-mobility group box 1 (HMGB1) was found to be over-expressed in many kinds of human cancer, which binds with several receptors and activates RAGE-Ras-MAPK, Toll-like receptors, NF-κB, and Src family kinase signaling pathways and plays a crucial role in tumorigenesis and cancer progression. However, the function and mechanism of HMGB1 in hepatocellular carcinoma (HCC) remain unclear. The aim of this study was to investigate the effect of HMGB1 on HCC progression and explore new molecular mechanism. HMGB1 transient knockdown, stable knockdown, and re-expression were performed by transfection with specific siRNA, shRNA, or expression vector in HCCLM3 cells. Results showed that transient knockdown HMGB1 prevented cell proliferation, promoted apoptosis, induced S phase arrest, and inhibited migration and invasion in vitro, and stable knockdown HMGB1 inhibited xenograft growth in Balb/c athymic mice in vivo. Molecular mechanism investigation revealed that knockdown HMGB1 significantly reduced the activation of MAPKs, including ERK1/2, p38, SAPK/JNK, as well as MAPKKs (MEK1/2, SEK1) and its substrates (c-Jun, c-Myc); downregulated NF-κB/p65 expression and phosphorylation level; decreased MMP-2 expression and activity; and upregulated p21 expression. Interestingly, c-Myc was firstly found to be involved in the promoting function of HMGB1 on HCC progression, which provided a novel clue for the inhibitory effect of HMGB1 on p21 expression by a p53-independent pathway. Collectively, these findings indicated that HMGB1 promoted HCC progression partly by enhancing the ERK1/2 and NF-κB pathways, upregulating MMP-2, and downregulating p21 via an ERK/c-Myc pathway.

**Electronic supplementary material:**

The online version of this article (doi:10.1007/s13277-015-4049-z) contains supplementary material, which is available to authorized users.

## Introduction

Hepatocellular carcinoma (HCC) is one of the most common and aggressive malignant tumors worldwide [1]. But the mechanisms of tumorigenesis and cancer progression of HCC are still unclear [2]. Therefore, to identify relevant genes and explore their function and mechanisms are crucial to the prevention and treatment of HCC. High-mobility group box 1 (HMGB1) is a non-histone chromosomal protein implicated in diverse biological processes, including DNA replication, extracellular signaling [3, 4], nucleosome stabilization, and transcriptional facilitation [5]. HMGB1 could also be a target for inflammation control [6].

Studies have shown that HMGB1 is over-expressed in many kinds of cancer tissues, including breast [7], lung [8], colon [9], nasopharyngeal [10], prostate [11], hepatocellular carcinoma [12], and melanoma [13]. HMGB1 was located both in mucleus and cytosol, also secreted into extracellular space [14]. Nuclear HMGB1 binds to DNA and interacts with various transcription factors, including the NF-κB members, p53, and TATA-binding proteins [15–17]. The cytoplasmic HMGB1 was found to bind with a number of molecules related to cancer progression, including factors involvement in cell cycle progression, cell proliferation, and anti-apoptosis [18, 19].

The extracellular HMGB1 can bind with high affinity to several receptors. It is well documented that HMGB1 binds to the receptor for advanced glycation end products (RAGE) and activates the Ras-MAPK pathway, which results in expression of MMP-2 and MMP-9 [20–22]. Recent evidences revealed that HMGB1 bound to Toll-like receptor (TLR)-2, TLR-4, TLR-9, CD24, and CXCR4, leading to activation of multiple signaling pathways, including NF-κB, ERK, p38 MAPK, and Src family kinases [23–26]. Many studies suggested that HMGB1 interacts with RAGE mainly in tumor cells but not in normal tissues [27]. Blockade of HMGB1-RAGE-MAPK signaling has been demonstrated to suppress tumor growth and metastasis [28]. HMGB1-specific silencing significantly decreased gastric cancer cell MGC-803 proliferation by reducing cyclin D1 expression, sensitized cells to induce apoptosis, and significantly reduced cellular metastatic ability and MMP-9 expression [29]. The serum HMGB1 level in HCC is significantly higher than that in liver cirrhosis, chronic hepatitis, and healthy status [30]. HMGB1 released from hypoxic HCC cells could activate TLR-4 and RAGE signaling pathways, induce inflammation, and promote cancer invasion and metastasis [31]. Knockdown of HMGB1 in HCCLM3 cell inhibited cell proliferation, migration, and invasion as previously reported by other researchers [32]. However, the function and mechanism of HMGB1 in HCC remains unclear. HCCLM3 is a human hepatocellular carcinoma (HCC) cell line with a highly metastatic potential [4]. In the present study, the effects of HMGB1 on HCCLM3 cell growth and invasion were investigated, and the mechanisms involved were further examined.

## Materials and methods

### Cell line and animals

HCCLM3 cell was derived from HCC cell lines (MHCC97) with high metastatic potential and provided by Liver Cancer Institute and Zhong Shan Hospital of Fudan University. Nude mice (male BALB/c nu/nu, 4-week-old) were obtained from Shanghai SLAC Laboratory Animal Co., Ltd., Chinese Academy of Sciences, and maintained in accordance with Guidelines for the Care and Use of Laboratory Animals, as published by the National Academy Press.

### Cell culture, transfection, and real-time PCR analysis

HCCLM3 cells were grown in Dulbecco’s modified Eagle’s medium (DMEM, Thermo Scientific, USA) supplemented with 10 % fetal bovine serum (FBS, Biochrom, Germany) and maintained at 37 °C with 5 % CO_2_. Small interfering RNA (siRNA) oligonucleotides against HMGB1 and the scrambled sequences were synthesized by RiboBio Company (Guangzhou, China). The following siRNA sequences were used: siHMGB1-1, 5′-GGAGGAAGAUGAAGAAGAUdTdT-3′ (sense); siHMGB1-2, 5′-GGACAAGGCCCGUUAUGAAdTdT-3′ (sense). HMGB1 was knocked down by specific siRNA (50 nM) transfections using Lipofectamine 2000 (Invitrogen, USA) according to the manufacturer’s protocol. At 48 h after transfection, cells were collected for RNA and protein extraction. Total RNA was extracted using TransZol UP (TransGen Biotech, China). Reverse transcription was performed using PrimeScript RT reagent Kit (TaKaRa, Japan). Quantitative real-time PCR analyses were performed by Applied Biosystems (7500 system) using SYBR Premix Ex Taq^™^ (TaKaRa, Japan) according to the manufacturer’s instructions. β-Actin gene was used as the internal control. The primers were as follows: HMGB1, 5′-TGCTCAGAGAGGTGGAAGACCA-3′ (forward) and 5′-TTGGGCGATACTCAGAGCAGAA-3′ (reverse); β-ACTIN, 5′-GGACTTCGAGCAAGAGATGG-3′ (forward) and 5′-AGCACTGTGTTGGCGTACAG-3′ (reverse). The relative expression of HMGB1 was calculated using 2^−ΔΔCt^ method. Expression analysis was performed in triplicate for each sample.

### Western blot analysis

The whole-cell extracts were prepared using RIPA lysis buffer (Beyotime, China) with phenylmethanesulfonyl fluoride and protease inhibitor cocktail (Roche, USA) and subjected to 10 % sodium dodecyl sulfate-polyacrylamide gelelectrophoresis (SDS-PAGE), with 30 μg of load per lane. Then, the membranes adhered with proteins were incubated with primary antibodies (Supplemental Table 1) overnight at 4 °C and probed with the corresponding horseradish peroxidase-conjugated secondary antibodies (KPL, USA). Chemiluminescence detection of membranes was conducted with ECL detection system (GE, RPN2132) and imaged under Las4000 Luminescent Imaging Analyzer (BioRad). Densitometry was performed using ImageJ software. The value of density ratio (target protein/β-actin) represented the relative level of protein expression. For the western blot analysis of xenograft tumors, tissue samples were initially homogenized and lysed in RIPA lysis buffer at 4 °C for 1 h, then centrifuged at 15,000 rpm for 15 min at 4 °C. And the supernatants were subjected to western blot analysis. The experiment was performed in triplicate.

### HCCLM3 cell proliferation assays

To determine the effect of HMGB1 on HCCLM3 cell growth in vitro, cells were plated at a density of 2.5 × 10^3^ cells/well in 96-well plates. After 24-h culture, siRNAs specific to HMGB1 and negative control were transfected into the cells at a density of 50 nM/well and six parallel wells for each siRNA. At the followed 1, 2, 3, 4, and 5 days post-transfection, cell viability was assessed using cell counting kit-8 (CCK-8, Dojindo, Japan) according to the manufacturer’ s instruction; then, the absorbance was read at 450 nm using microplate reader (BioTek, USA). The experiment was performed in triplicate.

### Analysis of cell cycle by flow cytometry

To determine the effect of HMGB1 on cell cycle, HCCLM3 cells were plated in 24-well plates at a density of 7.5 × 10^4^ cells/well. After 24-h culture, siRNAs specific to HMGB1 and negative controls were transfected into the cells at the density of 50nM/well and three parallel wells for each siRNA. After 72-h culture, the cells were collected and centrifuged at 200×*g* for 5 min. Cell pellets were re-suspended in 500 μL of ice-cold 70 % ethanol and fixed for at least 24 h at −20 °C. Then, the fixed cells were centrifuged at 500×*g* for 5 min and re-suspended in phosphate buffered saline (PBS) containing ribonuclease A and stained with propidium iodide (PI) for 30 min at room temperature. The percentage of cells in G1, S, and G2/M phases of the cell cycle was analyzed by flow cytometry.

### Analysis of apoptosis by flow cytometry

To determine the effect of HMGB1 on HCCLM3 cell apoptosis, we knocked down HMGB1 by specific siRNA transfections as described above. At 48 h after transfection, cells were collected and analyzed using Annexin V-FITC apoptosis detection kit (BioVision, USA). In brief, cells were washed twice and re-suspended at the density of 5 × 10^5^ cells/100 μL in binding buffer with 5 μL of PI and 5 μL of Annexin V-FITC. After incubation at room temperature for 5 min in dark, cells were subjected to flow cytometry for analysis of apoptosis. The cells only stained with Annexin V-FITC (FL1) were in the early stage of apoptosis; those positive for both Annexin V-FITC and PI (FL2) were in the stage of late apoptosis. Experiments were performed in triplicate.

### Analysis of cell migration and invasion ability

Migration assay was performed in a 24-well transwell chamber (BD, USA) containing a polycarbonate membrane filter (pore size, 8 μm) without Matrigel coating. Approximately 8 × 10^4^ cells/insert were suspended in DMEM without FBS, and the medium supplemented with 20 % FBS was added to the bottom chamber. After 48 h, the transwell chambers were fixed with 4 % paraformaldehyde and stained with crystal violet. The invasion assay was conducted in a similar manner but with 45 μg/50 μL Matrigel precoating on the filters and culture time for 72 h. The number of trans-membrane cells was counted under randomly selected five fields per well using microscope. The experiment was performed in triplicate.

### Construction of stable cell lines

HMGB1 was stably suppressed by the vector-based transfection of a specific shRNA (pMKO.1-shRNA) in HCCLM3 cell. Specific short hairpin RNA (shRNA) against HMGB1 was cloned into pMKO.1-puro retroviral vector to facilitate knockdown of HMGB1 expression. The shRNA target sequences (shHMGB) and negative control sequences (shNC) were listed as follows: shHMGB1-1, 5′-CCCAGATGCTTCAGTCAACTT-3′ (sense); shHMGB1-2, 5′-GGAGGAAGATGAAGAAGAT-3′ (sense); shNC1, 5′-CCTAAGGTTAAGTCGCCCTCG-3′ (sense); shNC2, 5′-TTCTCCGAACGTGTCACGT-3′ (sense). HCCLM3 cells were infected with retrovirus particles containing different shRNA sequences packaged from 293T cells, respectively, and the resistant cells were screened with puromycin. The HMGB1 stable knockdown cells were confirmed by testing HMGB1 expression through RT-qPCR and western blot. Furthermore, HMGB1 was re-expressed by the vector-based transfection of full-length HMGB1 (pCDH-HMGB1) in its stable knockdown cells. Full-length human HMGB1 was amplified using PCR and cloned into pCDH-CMV-MCS-EF1-copGFP lentiviral vector between *Eco*RI and *Not*I sites. The primers for PCR are 5′-GTCCGAATTCACCACCATGGGCAAAGGAGATCCTAA-3′ (forward) and 5′-CGCCGCGGCCGCTTATTCATCATCATCATCTT-3′ (reverse). The constructed vectors were verified by sequencing. Lentiviral partials containing HMGB1 obtained from 293T package cells were added to HMGB1 stable knockdown cells. Culturing for several days, a majority of cells were observed to emit green fluorescence under fluorescence microscope for GFP expression (Supplementary Figure S1), then subjected to flow cytometry for selecting the cells with green fluorescence. The re-expression of HMGB1 in the stable knockdown cells was analyzed by western blot.

### Tumor formation assay in nude mice

To investigate the effect of HMGB1 on HCC growth in vivo, we generated xenograft subcutaneous tumors in nude mice. The mice were randomly divided into four groups with five differently marked mice in each group. HMGB1 stable knockdown cell lines (shHMGB1-1 and shHMGB1-2) and control cell lines (shNC1 and shNC2) were respectively injected into nude mice. In brief, 5 × 10^6^ HCCLM3 cells in 200 μL of PBS were subcutaneously injected into the right flank of mice by using a 1-mL syringe needle. After palpable tumors were formed, the tumor sizes were measured every 5 days (tumor volume = *LW* ^2^/2 and plotted in mm^3^, where *L* is the length and *W* is the width, i.e., the longest and shortest perpendicular diameters of tumors, respectively). Tumor weights were determined at the 35th day, and the tumor growth curve was drawn. HMGB1 expression level in subcutaneous tumor was detected by western blot analysis.

### MMP-2 activity assay by gel zymography

Approximately 20 μg protein of each sample was loaded into different lanes of 10 % SDS-PAGE gel containing 1 mg/mL gelatin. After electrophoresis, the gel was washed twice with elution buffer (2.5 % Triton X-100, 50 mmol/L Tris-HCL, 5 mmol/L CaCl_2_, and 1 μmol/L ZnCl_2_; pH 7.6) for 1 h at room temperature to remove SDS. Then, the gel was washed twice with washing buffer (50 mmol/L Tris-HCl, 5 mmol/L CaCl_2_, and 1 μmol/L ZnCl_2_; pH 7.6) for 40 min and incubated at 37 °C in the reaction buffer (50 mmol/L Tris-HCL, 5 mmol/L CaCl_2_, 1 μmol/L ZnCl_2_, and 0.02 % Brij-35; pH 7.6) for 48 h. After the gel was stained with 0.05 % Coomassie brilliant blue, MMP activity was identified as a clear band against blue background.

### Statistical analysis

Values were expressed as mean ± SD. Student’s *t* test was used to determine significant difference between compared groups. *P* < 0.05 indicated significant difference.

## Results

### Downregulation of HMGB1 inhibits HCCLM3 cell proliferation

**Fig. 1:**
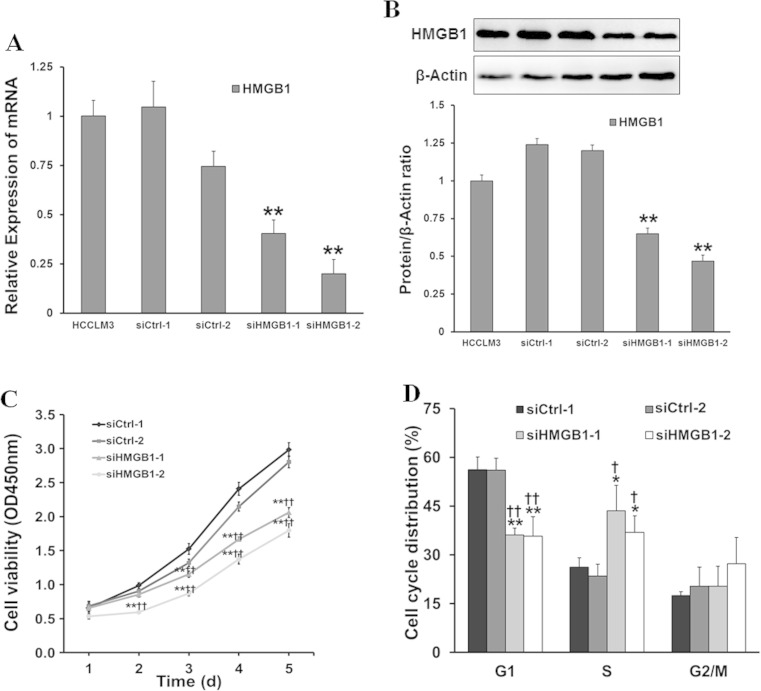
Knockdown of HMGB1 inhibited HCCLM3 cell proliferation and induced S phase arrest. HMGB1 knockdown in HCCLM3 cells was performed by transfection of siRNA specific targeting at HMGB1 (*siHMGB1*) or non-targeting negative control (*siCtrl*). **a**, **b** The interference effect was detected by real-time PCR for mRNA and western blot for protein expression. **c** The cell viability was measured by cell counting kit-8 assay at 1, 2, 3, 4, and 5 days after transfection, and cell growth was analyzed between cells with or without HMGB1 knockdown. **d** Cell cycle was tested by flow cytometry analysis. All statistical analysis was based on three independent experiments. *vs. siCtrl-1, *P* < 0.05; **vs. siCtrl-1, *P* < 0.01; ^†^vs. siCtrl-2, *P* < 0.05; ^††^vs. siCtrl-2, *P* < 0.01

HMGB1 knockdown was performed by transfecting specific HMGB1-siRNAs into HCCLM3 cells. The mRNA and protein expression level of HMGB1 evaluated by real-time PCR and western blot were significantly reduced in HCCLM3 cells transfected with siHMGB1-1/2 compared with the siCtrl-1/2 (Fig. 1a, b). To determine whether or not HMGB1 knockdown elicits an inhibitory effect on HCCLM3 cell proliferation, we analyzed cell growth by conducting the CCK-8 assay. The growth of cells with HMGB1 knockdown was significantly inhibited compared with that of controls (Fig. 1c). This result suggested that HMGB1 would play a key role in cell proliferation.

### HMGB1 knockdown inhibited cell proliferation and induced S phase arrest

**Fig. 2:**
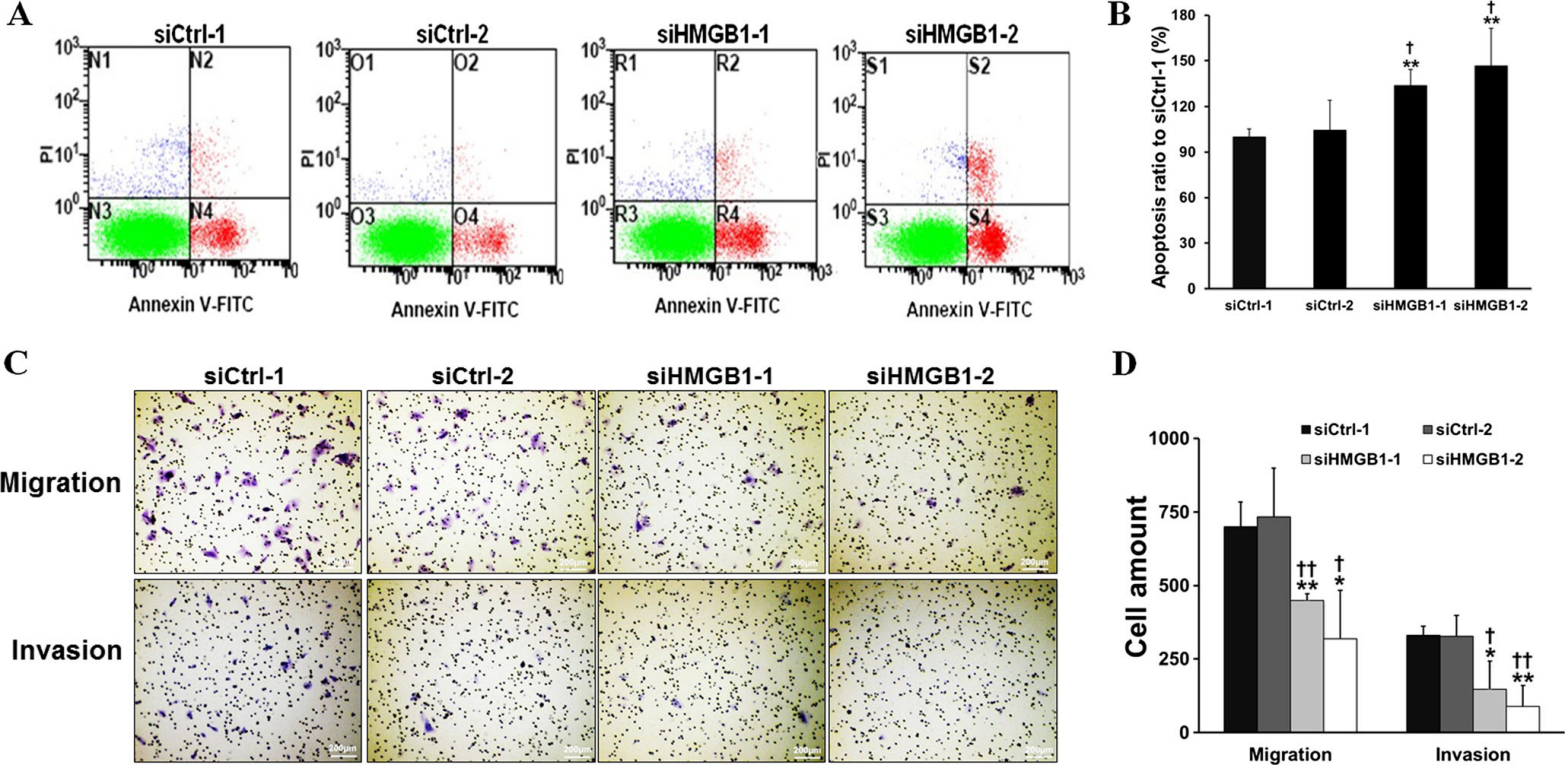
Knockdown of HMGB1 promoted HCCLM3 cell apoptosis and inhibited cell migration and invasion. **a** Cell apoptosis was tested by flow cytometry analysis using Annexin V/PI double staining method. **b** Cell apoptosis analysis was carried out using flow cytometry in cells with HMGB1 knockdown or control ones. **c** Trans-membrane cells was stained by crystal violet and observed under microscope (×100). **d** Cell migration activity was determined by counting trans-membrane cells in the inserts without Matrigel precoating, and cell invasion was measured by the same way but with Matrigel precoating inserts. The experiments were performed in triplicate. *vs. siCtrl-1, *P* < 0.05; **vs. siCtrl-1, *P* < 0.01; ^†^vs. siCtrl-2, *P* < 0.05; ^††^vs. siCtrl-2, *P* < 0.01

We further examined the effect of HMGB1 knockdown on cell cycle and cell apoptosis using flow cytometry. And the results showed that HMGB1 knockdown induced S phase arrest (Fig. 1d) and promoted cell apoptosis (Fig. 2a, b) in HCCLM3 cells. These results indicated that HMGB1 exhibited an important function in the regulation of S phase cell cycle transition and cell apoptosis.

### HMGB1 knockdown inhibits HCCLM3 cell migration and invasion

Transwell assay results revealed that cell migratory and invasive ability were lower in HMGB1-knocked down cells compared with those in control ones (Fig. 2c, d), which suggested that HMGB1 may promote cell migration and invasion in vitro.

### Stable HMGB1 knockdown inhibits the growth of xenograft tumor in vivo

**Fig. 3:**
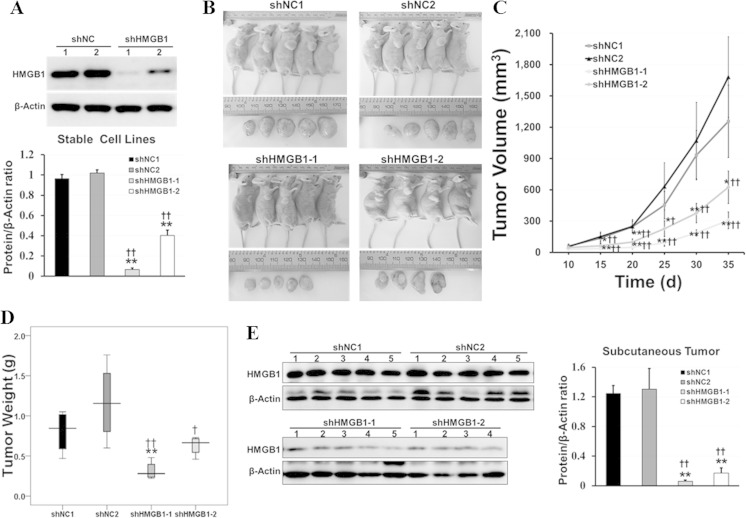
Stable knockdown of HMGB1 suppressed tumor growth in vivo. The effect of HMGB1 on HCC growth in vivo was performed by xenograft subcutaneous injection of stable HMGB1 knockdown cells or control ones in nude mice. Size and weight of subcutaneous tumors were measured after implantation, and HMGB1 expression in subcutaneous tumors was detected by western blot. **a** The expression of HMGB1 in cells with shNC or shHMGB1 was detected by western blot. **b** The pictures of nude mice and subcutaneous tumors were taken at the 35th day after implantation. **c** The size of subcutaneous tumors was measured every 5 days after implantation, and growth curve was draw after 35 days. **d** Weight of the subcutaneous tumors was measured at the 35th day after implantation. **e** HMGB1 expression in subcutaneous tumors from experimental and control groups was detected by western blot. *shNC1/2* HCCLM3 stable cell lines with control shRNA used as control group, *shHMGB1-1/2* HCCLM3 cell stable knockdown HMGB1 used as experimental group. Statistical significance was determined by two-tailed Student’s *t* test with SPSS software. *vs. shNC1, *P* < 0.05; **vs. shNC1, *P* < 0.01; ^†^vs. shNC2, *P* < 0.05; ^††^vs. shNC2, *P* < 0.01

The shRNAs specific of HMGB1 or negative control were cloned into pMKO.1, and two stable HMGB1-knocked down cell lines (shHMGB1-1 and shHMGB1-2) and two control cell lines (shNC1 and shNC2) were constructed. Western blot assay demonstrated that HMGB1 expression was suppressed in stable knockdown cells as showed in Fig. 3a. To investigate the biological effect of HMGB1 on HCC growth in vivo, we generated xenograft subcutaneous tumors in nude mice. As expected, the mice injected with HMGB1 knockdown cells (shHMGB1-1 and shHMGB1-2) developed smaller solid tumors than those injected with the control cells (shNC1 and shNC2; Fig. 3b). The stable knockdown of HMGB1 in HCCLM3 cells resulted in slower growth of xenograft in Balb/c athymic mice in tumor size (Fig. 3c) and weight (Fig. 3d) than those of the control ones (Supplemental Table 2). The inhibitory role of shHMGB1-1 was more effective than that of shHMGB1-2 which was consistent with the RNA interference efficiency of shHMGB1-1 stronger than that of shHMGB1-2 (Fig. 3a). Western blot analysis revealed that HMGB1 expression was significantly suppressed in the stable knocked down cells (Fig. 3a) and subcutaneous tumor generated from these cells (Fig. 3e), which is consistent with the effect in vitro. These results demonstrated that HMGB1 is a critical modulator of the growth of xenograft tumors in nude mice.

### Effect of HMGB1 knockdown on MAPKs and NF-κB expression and phosphorylation

**Fig. 4:**
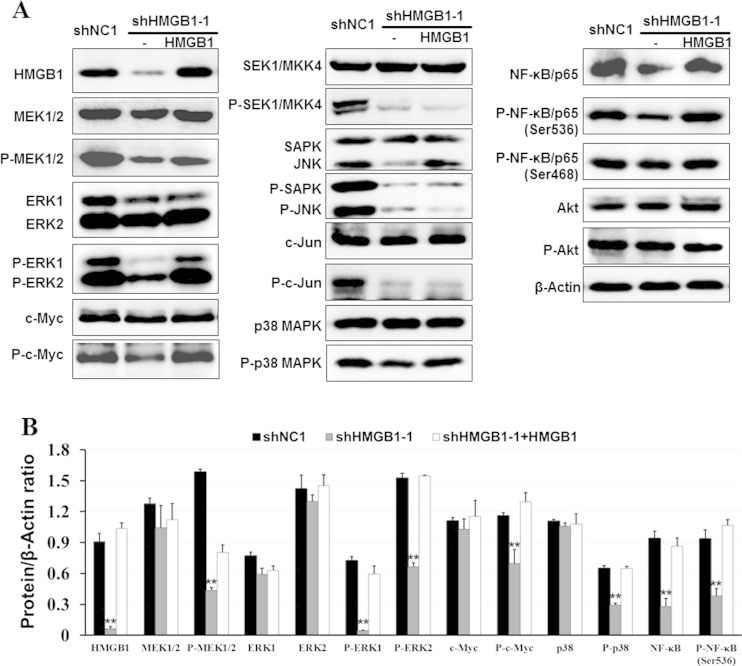
Stable knockdown and re-expression of HMGB1 affected protein expression and phosphorylation levels of MAPKs and NF-κB. HMGB1 re-expression was performed by transfection of pCDH-HMGB1 in the HMGB1 stable knockdown HCCLM3 cell line, and protein expression was detected by western blot. All experiments were performed in triplicate. **a** Protein expression and phosphorylation levels of members of MAPKs and NF-κB signaling pathways. **b** Densitometric analysis for protein bands relative to β-ACTIN using ImageJ software. **vs shNC1 or HMGB1 re-expression, *P* < 0.01

To further investigate the downstream molecules of HMGB1, we performed western blot to examine the expression and phosphorylation of proteins in several important signaling pathways, e.g., MAPKs and NF-κB/p65. HMGB1 knockdown reduced the activation of MAPKs, including ERK1/2, p38, and SAPK/JNK, as well as MAPKKs (MEK1/2, SEK1) and substrates (c-Jun, c-Myc). HMGB1 knockdown not only downregulated the expression of NF-κB/p65 but also decreased its phosphorylation level at Ser536. By contrast, re-expression of HMGB1 in the knocked down cells enhanced the phosphorylation levels of MEK1, ERK1/2, p38, c-Myc and NF-κB/p65, except SEK1, SAPK/JNK, and c-Jun (Fig. 4a, b).

### HMGB1 stable knockdown upregulates p21 and decreases MMP-2 activity

**Fig. 5:**
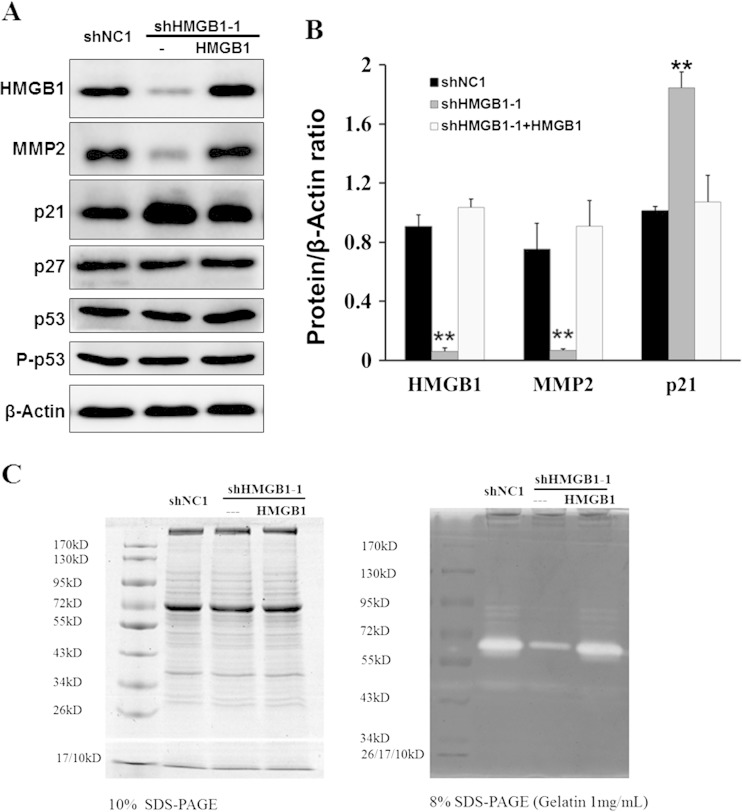
Stable knockdown and re-expression of HMGB1 affected p21 expression and MMP2 expression and activity. **a** The expression of HMGB1, MMP2, p21, p27, p53, and P-p53 in HMGB1 knockdown and re-expression cells were detected by western blot. **b** Densitometric analysis for HMGB1, p21, and MMP2 protein bands relative to β-actin was analyzed using ImageJ software. **c** MMP2 activity was determined by gel zymography, and the protein quantity was measured by SDS-PAGE and Coomassie blue staining. **vs shNC1 or HMGB1 re-expression, *P* < 0.01

HMGB1 knockdown results in S phase arrest and suppression of cell migration and invasion, so we investigated the expression of several key cell cycle inhibitors and MMP-2 expression and activity affected by HMGB1. The results showed that p21 was upregulated in stable HMGB1 knockdown HCCLM3 cells but downregulated in HMGB1 re-expressed cells (Fig. 5a, b). By comparison, the expression and phosphorylation levels of p53 and p27 were not affected by HMGB1. Furthermore, zymography assay showed that the MMP-2 activity in HCCLM3 cells was decreased as HMGB1 was knocked down, but this activity was enhanced as HMGB1 was re-expressed in stable knockdown cells (Fig. 5c). Each lane was loaded with the same amount of protein sample as verified by SDS-PAGE and Coomassie blue staining (Fig. 5c). Western blot data confirmed the downregulation of MMP-2 in stable HMGB1 knockdown cells (Fig. 5a, b).

## Discussion

In this study, the effect of HMGB1 on HCCLM3 cell proliferation and invasion as well as the mechanisms involved was determined. We found that knockdown of HMGB1 in HCCLM3 cells by siRNA resulted in growth inhibition, apoptosis improvement, and S phase cell cycle arrest. This process also inhibited cell migration and invasion in vitro, and stable knockdown of HMGB1 in HCCLM3 cells inhibited the growth of xenograft in Balb/c athymic mice in vivo.

However, the mechanism of HMGB1 in tumorigenesis and tumor progression is partially understood. Studies have shown that HMGB1 over-expression is associated with self-sufficiency in growth signals and insensitivity to growth inhibitors, mainly via AKT, MAPKs, and NF-κB pathways [4]. HMGB1-RAGE interaction may activate NF-κB, PI3K/AKT, and MAPK signaling pathways [29, 33]. To understand whether or not the function of HMGB1 in HCC is dependent of MAPKs, NF-κB and AKT signaling pathways, we analyzed the effect of alteration of HMGB1 expression on activity of those pathways in HCCLM3 cells. Consistent with former studies [31–34], we also found that the expression and phosphorylation levels of NF-κB/p65 and the activation of MAPKs, including ERK1/2, p38, SAPK/JNK, MEK1/2, SEK1, c-Jun, and c-Myc, were regulated by HMGB1. Because these molecules have been verified to participate to cell proliferation, apoptosis and cell cycle control. consequently, which could be reasonable explicated the phenotypic changes about cell proliferation, apoptosis and cell cycle in HCCLM3 cells on account of HMGB1 knockdown. Fortunately and firstly, we found that c-Myc phosphorylation level varied along with the expression of HMGB1. C-MYC is a transforming oncogene, which is usually over-expressed in many kinds of human cancers. Resent study described that MT-MC1 and HMG1, two direct target genes of c-Myc, could each recapitulate multiple c-Myc phenotypes manipulated in c-Myc nullizygous cells, which indicated that HMGB1 is one of key functional target genes modulated by c-Myc [35]. But, whether adverse regulation exists is not clear. Our founding suggested that HMGB1 could regulate c-Myc phosphorylation in HCCLM3 cells, while partly for the activation of ERK1/2 or p38 regulated by HMGB1 contributes to c-Myc phosphorylation.

Cell cycle analysis showed that HMGB1 knockdown induced HCCLM3 cells S phase arrest, so the expressional alteration of several S phase inhibitors were detected after HMGB1 knockdown. The result showed that p21^*waf*/*ci1*^ (CDK inhibitor 1, CDKN1A, CKIp21) was upregulated in HMGB1 knockdown cells and downregulated when HMGB1 was re-expressed, while the expression and phosphorylation level of p53 and p27 were not changed. p21 is a transcriptional target of p53 and exhibits a crucial function in cell cycle arrest. The activation and suppression of p21 are usually regulated by p53-dependent or p53-independent modes [36, 37]. Considering that p53 was deficient in the HCC97 cell lines for its 249 codon mutation, and the expression and phosphorylation levels of p53 were not changed in HMGB1 knockdown cells [38, 39]. In addition, recent study reported that HMGB1 could control cell cycle association with p21 (Waf1/Cip1) via a p53-independent, Sp1-dependent pathway in melanoma [40]. Accordingly, the regulation mechanism of HMGB1 to p21 may be via a p53-independent mode in HCCLM3 cells. Many studies have revealed that c-Myc is an important negative regulator of p21 [41]. Myc-mediated antagonism of p21 is accomplished by the interaction of Myc with several proteins (such as KDM5B, DMNT3A, AP2C, Miz1) at the proximity of the TSS of CDKN1A that results in transcriptional repression [41–45]. And another mechanism via Ras pathway. In some chronic myeloid leukemia (CML) cell lines, Ras upregulates p21 expression, and activation of the p21 promoter by Ras was dependent on Sp1/3 binding sites. c-Myc antagonized the induction of p21 mediated by Ras by affecting Sp1 transcriptional activity [46]. In the present study, HMGB1 knockdown reduced the activation of c-Myc, which may fail to inhibit p21 transcription; as a result, p21 is upregulated. p21 could directly inhibit the activity of cyclin/cdk2 complexes and arrest cell cycle in S phase. Therefore, HMGB1 maybe control cell cycle partly associated with p21 via the ERK/c-Myc pathway.

Our work also found that HMGB1 knockdown in HCCLM3 inhibited cell migration and invasion in vitro. It is well known that MMP exhibits an important function in tumor invasion and metastasis by degrading the extracellular matrix. The extracellular HMGB1 could activate RAGE-Ras-MAPK pathway, which results in expression of MMP-2 and MMP-9 [47]. So, we further evaluated the expression and activity of MMP-2 in HMGB1 knockdown cells. The results showed that MMP-2 expression and activity decrease in HMGB1 knockdown cells and enhanced when HMGB1 was re-expressed. This result suggested that HMGB1 may be involved in HCCLM3 cell invasion by regulated expression and activity of MMP-2.

In conclusion, HMGB1 promotes the proliferation and invasion of HCCLM3 cells partly by enhancing ERK1/2 and NF-κB pathways, downregulating p21, and upregulating MMP-2. The inhibitory effect of HMGB1 on p21 expression may be p53 independent, via an ERK/c-Myc pathway.

## Electronic supplementary material

Below is the link to the electronic supplementary material.

Fig S1 (GIF 399 kb)

High Resolution (TIFF 1638 kb)

Table S1 (DOC 61 kb)

Table S2 (DOC 55 kb)
