# Supplementary figures and images for: HMGB1 promotes HCC progression partly by downregulating p21 via ERK/c-Myc pathway and upregulating MMP-2
Source: Tumour Biol. 2015 Oct 24;37(4):4399–408. doi: 10.1007/s13277-015-4049-z (PMC4844642; doi:10.1007/s13277-015-4049-z)

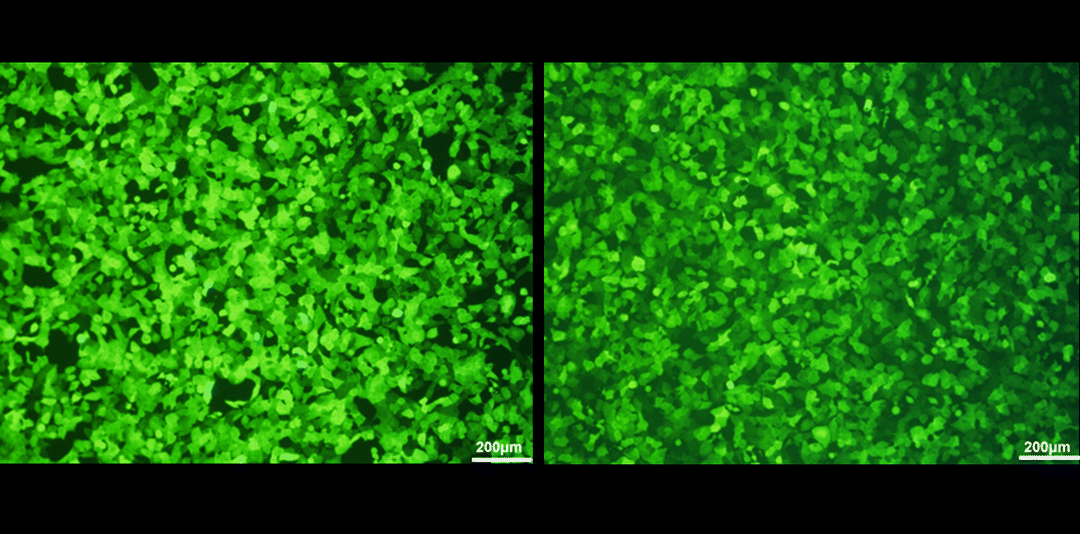

Supplement: Supplementary file 1 — (GIF 399 kb) [file 13277_2015_4049_Fig6_ESM.gif]

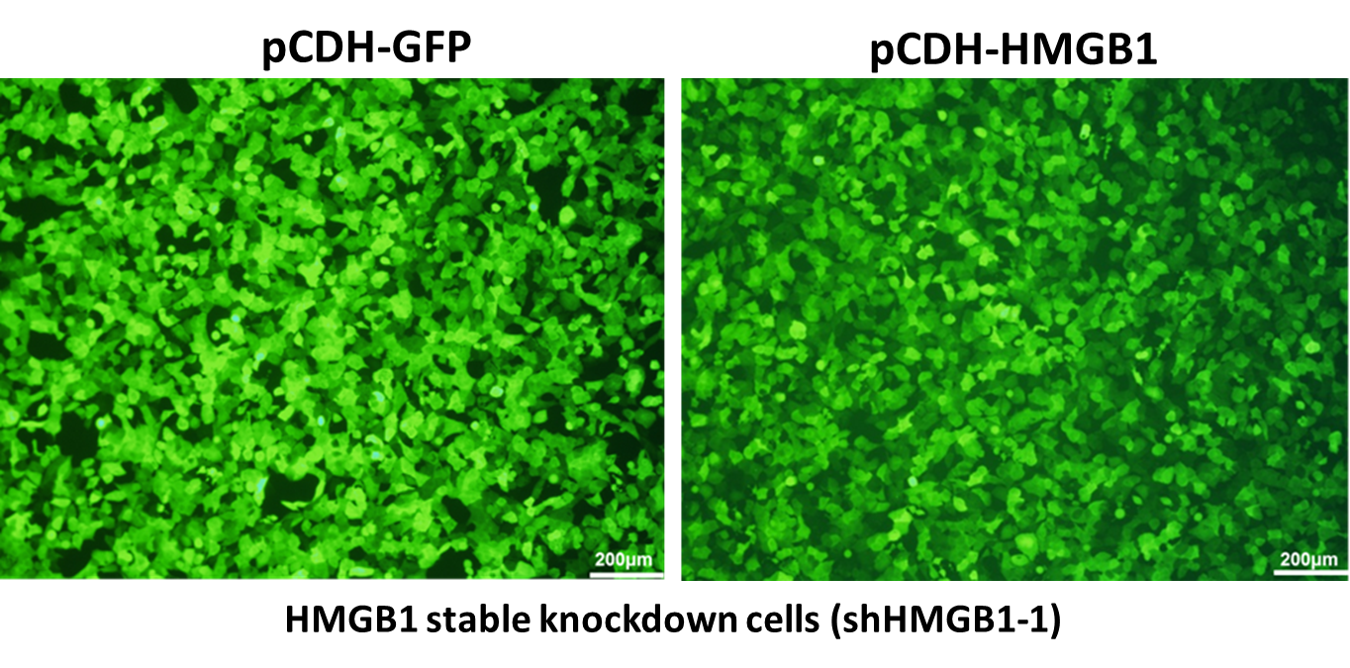

Supplement: Supplementary file 2 — High Resolution (TIFF 1638 kb) [file 13277_2015_4049_MOESM3_ESM.tif]
