# Supplementary material for: HMGB1 promotes HCC progression partly by downregulating p21 via ERK/c-Myc pathway and upregulating MMP-2
Source: Tumour Biol. 2015 Oct 24;37(4):4399–408. doi: 10.1007/s13277-015-4049-z (PMC4844642; doi:10.1007/s13277-015-4049-z)
Supplement: Supplementary file 3 — (DOC 61 kb) [file 13277_2015_4049_MOESM1_ESM.doc]

**Supplemental table 1**. The primary antibodies used in this study.

| **Antibody name** | **Company, country** |
| --- | --- |
| rabbit anti-p21 | Cell Signaling Company, USA |
| rabbit anti-p27 |
| rabbit anti-p53 |
| rabbit anti-phospho-p53 |
| rabbit anti-MEK1/2 |
| rabbit anti-phospho-MEK1/2 |
| rabbit anti-SEK1/MKK4 |
| rabbit anti-phospho-SEK1/MKK4 |
| rabbit anti-ERK1/2MAPK |
| rabbit anti-phospho-ERK1/2MAPK |
| rabbit anti-SAPK/JNK |
| rabbit anti-phospho-SAPK/JNK |
| rabbit anti-p38MAPK |
| rabbit anti-phospho-p38MAPK |
| rabbit anti-c-Jun |
| rabbit anti-phospho-c-Jun |
| rabbit anti-c-Myc |
| rabbit anti-Akt |
| rabbit anti-phospho-Akt |
| mouse anti-NF-κB/p65 |
| rabbit anti-phospho-NF-κB/p65 (Ser^536^) |
| rabbit anti-phospho-NF-κB/p65 (Ser^468^) |
| rabbit anti-HMGB1 | Abcam, USA |
| rabbit anti-Phospho-c-Myc |
| rabbit anti-MMP2 | Abgent, USA |
| mouse anti-β-actin | GenScript, China |
