# Supplementary material for: HMGB1 promotes HCC progression partly by downregulating p21 via ERK/c-Myc pathway and upregulating MMP-2
Source: Tumour Biol. 2015 Oct 24;37(4):4399–408. doi: 10.1007/s13277-015-4049-z (PMC4844642; doi:10.1007/s13277-015-4049-z)
Supplement: Supplementary file 4 — (DOC 55 kb) [file 13277_2015_4049_MOESM2_ESM.doc]

**Supplemental table 2. The final volume and weight of Xenograft Tumor in nude mice**.

|  | **Tumor volume (mm^3^)** | **Tumor weight (g)** |
| --- | --- | --- |
| shNC1 | 1256.90±347.60 | 0.85±0.26 |
| shNC2 | 1635.37±637.22 | 1.21±0.43 |
| shHMGB1-1 | 624.64 ±155.55**†† | 0.33±0.10**†† |
| shHMGB1-2 | 822.90 ±185.71*† | 0.63±0.12† |

*: *vs* shNC1, *P* < 0.05; **: *vs* shNC1, *P* < 0.01; †: *vs* shNC2, *P* < 0.05; ††: *vs* shNC2, *P* < 0.01
